# Ambient air pollution during pregnancy and offspring cerebral palsy

**DOI:** 10.1016/j.envint.2026.110201

**Published:** 2026-03-18

**Authors:** Haoran Zhuo, Beate Ritz, Jason G. Su, Roch A. Nianogo, Joshua Warren, Zeyan Liew

**Affiliations:** a Department of Environmental Health Sciences, Yale School of Public Health, New Haven, CT, United States; b Yale Center for Perinatal, Pediatric, and Environmental Epidemiology, Yale School of Public Health, New Haven, CT, United States; c Department of Epidemiology, Fielding School of Public Health, UCLA, Los Angeles, CA, United States; d Department of Neurology, School of Medicine, UCLA, Los Angeles, CA, United States; e School of Public Health, University of California, Berkeley, Berkeley, CA, United States; f Department of Biostatistics, Yale School of Public Health, New Haven, CT, United States

**Keywords:** Cerebral palsy, Ambient air pollution, Chemical mixture, Negative control

## Abstract

**Background:**

Cerebral palsy (CP) is a permanent neuromotor disorder with childhood onset, and the etiology for most cases remains unexplained.

**Methods:**

We conducted a population-based case-control study in California during 2000–2015, including all identified CP cases (N = 9,343) from the California’s Department of Developmental Services and a control group of 20% random sample of live births without CP (N = 1,560,423). We employed a high-resolution (100 m) spatiotemporal model to estimate prenatal exposures to fine particulate matter (PM_2.5_), nitrogen dioxide (NO_2_) ozone (O_3_), and investigated their impacts on CP through single-pollutant, multiple-pollutant, and chemical-mixture models. Further, we examined the unmeasured confounding bias through the negative control exposure (NCE) design and assessed the potential heterogeneity across CP subtypes.

**Findings:**

Prenatal exposures throughout pregnancy to ambient NO_2_ (per interquartile-range, OR = 1.14, 95% CI, 1.10–1.18) and O_3_ (OR = 1.08, 95% CI, 1.05–1.11) were associated with higher odds of CP in offspring. A stronger association emerged when three pollutants were modelled as a mixture (OR = 1.23, 95% CI, 1.17–1.29) especially in the first and third trimesters. Potential interactions were noted across pollutants, with associations for NO_2_ and O_3_ strengthening in multiple-pollutant adjusted models or within strata of low PM_2.5_ (i.e., below the median). PM_2.5_ was positively associated with CP only in strata of low O_3_ (OR = 1.14, 95% CI, 1.09–1.19). The 36 months post-birth NCE was not associated with CP, suggesting no strong confounding bias. CP involving ataxic and dyskinetic motor dysfunction was more sensitive to O_3_ exposure.

**Conclusions:**

Prenatal exposure to major ambient air pollutants was associated with offspring CP. Future studies to elucidate the underlying mechanisms are recommended.

## 1. Introduction

Cerebral palsy (CP) is a lifelong neuromotor disorder of childhood onset that permanently impairs motor function and muscle control in 2–3 children per 1000 live births (Colver et al., 2014). There was a modest decline in CP prevalence over the past two decades, likely due to advances in obstetric and neonatal care (Novak et al., 2025). Nevertheless, only a small fraction of CP was deemed to have definitive causes, such as hypoxic–ischemic brain injury and neonatal stroke, with the etiology for most cases unexplained (Korzeniewski et al., 2018). Research on environmental risk factors and CP has grown in recent years (Liew et al., 2020; Zhuo et al., 2023; Zhuo et al., 2025; Liew et al., 2020; Liew et al., 2014).

Ambient air pollution is arguably the most widespread environmental pollutant, with ~18% of neonatal disorders attributable to air pollution exposure every year (Murray et al., 2020). Several neurodevelopmental disorders like autism and intellectual disability have also been linked to prenatal air pollution exposure (Volk et al., 2021), while knowledge remains scarce for CP. Strong clinical predictors of CP, such as prematurity and restricted intrauterine fetal growth, have been consistently associated with prenatal exposure to ambient air pollutants (Guo et al., 2019). To date, only two recent Canadian studies have linked exposure to fine particulate matter (PM_2.5_) and the risk for CP (Zhang et al., 2024; Ahmed et al., 2025). However, these studies utilized a relatively spatially-coarse models to assess pollutants with high spatial heterogeneity (Zhang et al., 2024). Further research with enhanced exposure assessment and applies to a demographically diverse population is urgently needed.

We employed a sophisticated air pollution model with high spatial-temporal resolution in a statewide population-based study in California to evaluate the association between prenatal exposure to fine particulate matter (PM_2.5_), nitrogen dioxide (NO_2_), and ozone (O_3_) and CP risk in the offspring. We evaluated potential interactions among pollutants, heterogeneity in CP subtypes, and influence from unmeasured confounding through a negative control analysis.

## 2. Methods

### 2.1. Study design and population

We conducted a case-control study nested in our California cohort for Cerebral Palsy study (Cal-CP) (Zhuo et al., 2023) that includes over 8 million live births in California across sixteen years from 2000 to 2015. We included all linked CP cases and employed a cumulative sampling approach by including a 20% random sample from all live births without CP by the end of the study period as the control comparison. CP cases were ascertained using the diagnostic records from the California Department and Developmental Services (DDS) during 2000–2021. The DDS is a statewide referral and service system in California that provides diagnosis and supporting services to all residents with certain developmental disabilities without restrictions on financial or citizenship status (Department of Developmental Services, 2015). Validation studies have shown that motor function variables reported in DDS had high inter-rater reliability that exceeds 0.85 (Jamie and Harold, 2023).

The linkage between the California DDS and birth records was performed through a probabilistic linkage approach that has been described elsewhere (Zhuo et al., 2023). In brief, the record linkage was based on parents’ and children’s personal identifying information (name, date of birth, and sex of child) from both databases, and completed through the Linkplus software created by the Centers for Disease Control and Prevention. Probability-based linkage scores were generated for all identified pairs, and less certain linked pairs (e.g., lower linkage score) were manually checked by three researchers independently. The overall linkage rate for CP records is 93%, with non-linked pairs mainly due to missing linkage information. We found comparable characteristics in mothers and children for non-linked and linked records with respect to maternal age at delivery, primary language spoken, sex of the child, and the child’s birth year (Zhuo et al., 2025). Further, we excluded observations with missing maternal residential address at delivery on the birth records, unreasonable values on gestational age (<22 or >44 week) that were susceptible to recording errors, and missing exposures throughout the pregnancy, generating the final analytical sample of 9,343 CP cases and 1,560,423 controls.

### 2.2. Outcomes

CP diagnosis in the DDS was defined as nonprogressive lesions or disorders in the brain characterized by paralysis, spasticity, or abnormal movement or posture control that manifested in early childhood (Department of Developmental Services, 2015). Several clinical subtypes of CP based on motor dysfunction were recorded, including spasticity reflecting increased muscle tension, ataxia reflecting disturbances in postural balance and coordination of muscle activity, dyskinesia reflecting involuntary movements, and mixed types with more than one motor dysfunction manifesting. Additionally, the DDS records reported the severity of motor dysfunction (mild, moderate, severe) based on the level of daily activities and functions, as well as the body area affected (unilateral or bilateral).

### 2.3. Ambient air pollution assessment

We geocoded the maternal residential addresses at delivery extracted from the birth records using the ArcGIS StreetMap Premium North America. We linked our point-address level geocodes to our newly developed high-resolution (100 meters) daily land-use regression (LUR) spatiotemporal model for ambient air pollutants of PM_2.5,_ NO_2_, and O_3_ in California (Su et al., 2024). Details of the LUR model have been presented elsewhere (Su et al., 2024). In brief, this model utilized the Deletion/Substitution/Addition (D/S/A) integrated LUR modeling technique, a machine learning (ML) algorithm that combines the strengths of both LUR and ML models to provide better prediction accuracy and flexibility. The modeling approach integrated information from daily traffic, bi-weekly vegetation dynamics, one-time land use and land cover specifics, daily meteorological conditions, daily measured remote sensing data, and other relevant factors. The model was trained using a ten-fold cross-validation on air pollution measurement data from government regulatory monitoring, fixed site saturation monitoring, and Google Streetcar mobile monitoring to enhance the spatiotemporal coverage, achieving a high coefficient of cross-validated determination (*R*^2^) for NO_2_ (0.84), O_3_ (0.93), and PM_2.5_ (0.65). From the modelled daily exposures, we calculated the pregnancy-averaged exposure levels from the date of birth to the last menstrual period recorded in the birth records, as well as trimester-specific exposure estimates (first trimester: day 0–97, second trimester: day 98–195, third trimester: day 196 to delivery) (Nguyen and Wilcox, 2005). Our exposure estimate was based on maternal residential address at delivery and did not account for residential mobility during pregnancy. However, prior studies showed that mothers tend to move locally, and the non-differential exposure misclassification bias for the overall association is expected to be small (Bell and Belanger, 2012).

## 3. Statistical analyses

### 3.1. Pregnancy and trimester-specific exposures

We first used logistic regression to estimate odds ratios (OR) and 95% confidence intervals (CIs) for CP according to per interquartile range (IQR) increase in PM_2.5_, NO_2_, or O_3_ separately as single-pollutant models. For estimating the trimester-specific exposure effect, we co-adjusted the exposure values of the three trimesters simultaneously. To consider the effects of multiple pollutants, we employed three approaches of 1) co-adjusted for all three pollutants simultaneously, 2) analyzed two-by-two interactions among pollutants through stratified analyses of one pollutant across the higher or lower levels of another pollutant using the median as a cut-off (e.g., per IQR increase of PM_2.5_ by O_3_ concentration below or above the median) and assessed the p-values for interactions, 3) examined the overall effect of all three pollutants as a mixture through the quantile-based g-computation method (R package qgcomp) (Keil et al., 2020).

In all regression models, we adjusted for an a *priori* list of maternal individual and neighborhood sociodemographic factors that may confound the associations between air pollution and CP presented in a directed acyclic diagram (eFig. 1). The covariates examined include maternal age at delivery (≤18, 19–25, 26–30, 31–35, >35 years), self-reported race/ethnicity [African American or Black, Asian, Hispanic or Latinx of any race, non-Hispanic White, Others], self-reported education level (<12th grade, high school or diploma, college and above), primary payment source for prenatal care (governmental programs, private, others), zip-code level rural-urban codes created by the USDA (USDA, 2025), and the census-tract level social vulnerability index (total SVI, continuous) created by the US CDC (Social Vulnerability Index, 2022). To address potential temporal confounding, we adjusted for the child’s exact birth year, and the season of conception (winter, spring, summer, fall).

Several confounders are examined in sensitivity analyses (eTable 1) due to their shorter availability (2007–2015), including maternal pre-pregnancy body mass index (BMI, kg/m^2^; obese (≥30)), overweight (25 to <30), normal weight (18.5 to <25), and underweight (<18.5)), cigarette smoking before and during pregnancy (yes, no), maternal receipt of Special Supplemental Nutrition Program (WIC) benefits during pregnancy (yes, no). We additionally adjusted for parity (1, 2, 3+) and the average ambient temperature during pregnancy generated from the Daymet (version 4, https://daymet.ornl.gov/).

### 3.2. Negative control exposure (NCE)

We performed an NCE analysis to inspect uncontrolled confounding bias. We generated and studied exposures starting from 36 months after delivery and over the exact gestational length (in days) as NCE, based on the same residential location at delivery as the index exposure. This window was selected because air pollution exposure after three years of age was unlikely to have a biological causal effect on congenital CP, and using the same residential address for both the index exposure and the NCE ensured a comparable spatial confounding structure (Colver et al., 2014; Lipsitch et al., 2010). An association between the NCE and CP would suggest the presence of unmeasured confounding bias (Lipsitch et al., 2010). We performed NCE analyses using the single-pollutant and mixture models, co-adjusted for the index exposure and the NCE.

### 3.3. Heterogeneity across clinical subtypes of CP

To investigate heterogeneity across CP phenotypes, we stratified the overall CP into spastic, ataxic, dyskinetic, or mixed CP. Further, we examined the severity of motor dysfunction (mild/moderate or severe) and the limbs affected (unilateral or bilateral) among spastic CP, the most common subtype. For these subgroup analyses, we focused on single-pollutant models that examine exposure over the entire pregnancy.

### 3.4. Sensitivity and secondary analyses

We conducted sensitivity analyses to examine the robustness of our main findings. First, we adjusted the additional potential confounding variables in our main models. Second, we used multiple imputation to calculate the missing values of the main covariates (<3%). Third, to address an on-average shorter gestational length of CP cases compared to the controls, we limited the prenatal exposure window to up to 32 gestational weeks. We also limited the analysis to term births only (≥ 37 gestational weeks) to facilitate comparison with previous studies (Zhang et al., 2024). Fourth, we excluded CP cases with postnatal accidents (including drowning, automobile, other types of vehicles, and other types of accidents) recorded in the DDS (Department of Developmental Services, 2015), given that these cases were less likely to have prenatal etiology.

Additionally, we examined the potential mediating effects through preterm delivery (<37 weeks or <32 weeks), low birth weight (defined as <2500 grams), and small for gestational age (defined as birth weight <10th percentile at each gestational age) (Nelson and Blair, 2015). We explored the heterogeneity by child’s sex, maternal socioeconomic status (race/ethnicity, educational levels, primary payment sources), and the neighborhood social vulnerability indexes (the total and the socioeconomic-domain scores).

The study protocol was approved by the official institutional review board at Yale University (IRB no. 2000028297) and the California Committee for the Protection of Human Subjects (project no. 2020–174). The study was exempted from the informed consent requirement because there was no contact with human subjects. We reported all findings following the Strengthening and Reporting of Observational Studies in Epidemiology (STROBE) guidelines.

## 4. Results

In our study, CP cases were more likely to be male, and born to mothers who were older at the time of pregnancy (>35 years old), self-identified as African American/Black or Hispanic/Latinx of any race, with lower educational attainment, utilized government insurance for prenatal care, or resided in neighborhoods with greater vulnerability compared to controls (Table 1). The median exposure levels of PM_2.5_, NO_2_, and O_3_ were slightly higher among CP cases (eTable 2). We observed moderate positive correlations between NO_2_ and PM_2.5_ (r = 0.40–0.43) while negative correlations were noted for O_3_ with NO_2_ and PM_2.5_ (r = −0.38 to −0.12) (eTable 3).

**Fig. 1.**
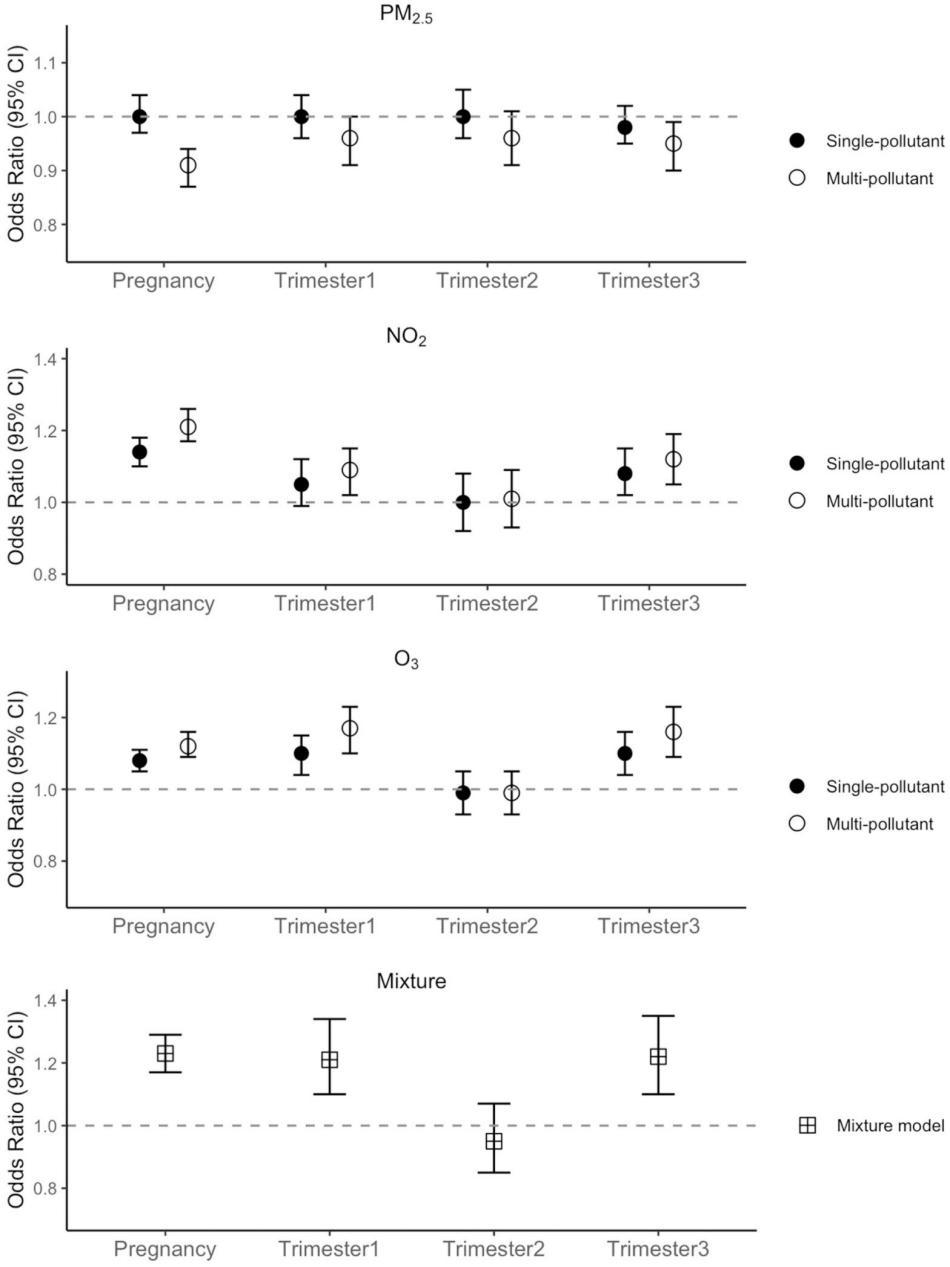
Associations between ambient particulate matter with a diameter 2.5 μm or smaller (PM_2.5,_ in ug/m^3^), nitrogen dioxide (NO_2_, in ppb), ozone (O_3_, in ppb) during pregnancy and the risk of cerebral palsy, modeled as single pollutant (solid dots), multiple pollutants (hollow dots), and as a chemical mixture (matts dots). Estimated odds ratio (ORs) and 95% confidence intervals (CIs) of cerebral palsy for ambient air pollution at per interquartile range (IQR) increase, adjusted for year of birth, season of conception, maternal individual characteristics (age at delivery, race/ethnicity, education level, primary insurance type of prenatal care), zip-code level urban rural commuting code, and census-tract social vulnerability index (total score). Trimester-specific exposures were co-adjusted in the same model. Single-pollutant model assessed each pollutant separately, multiple-pollutant model was modelled by co-adjusting all three pollutants in the same model, mixture model was fitted use quantile g-computation method, treating all three pollutants as a chemical mixture in each exposure window.

**Fig. 2.**
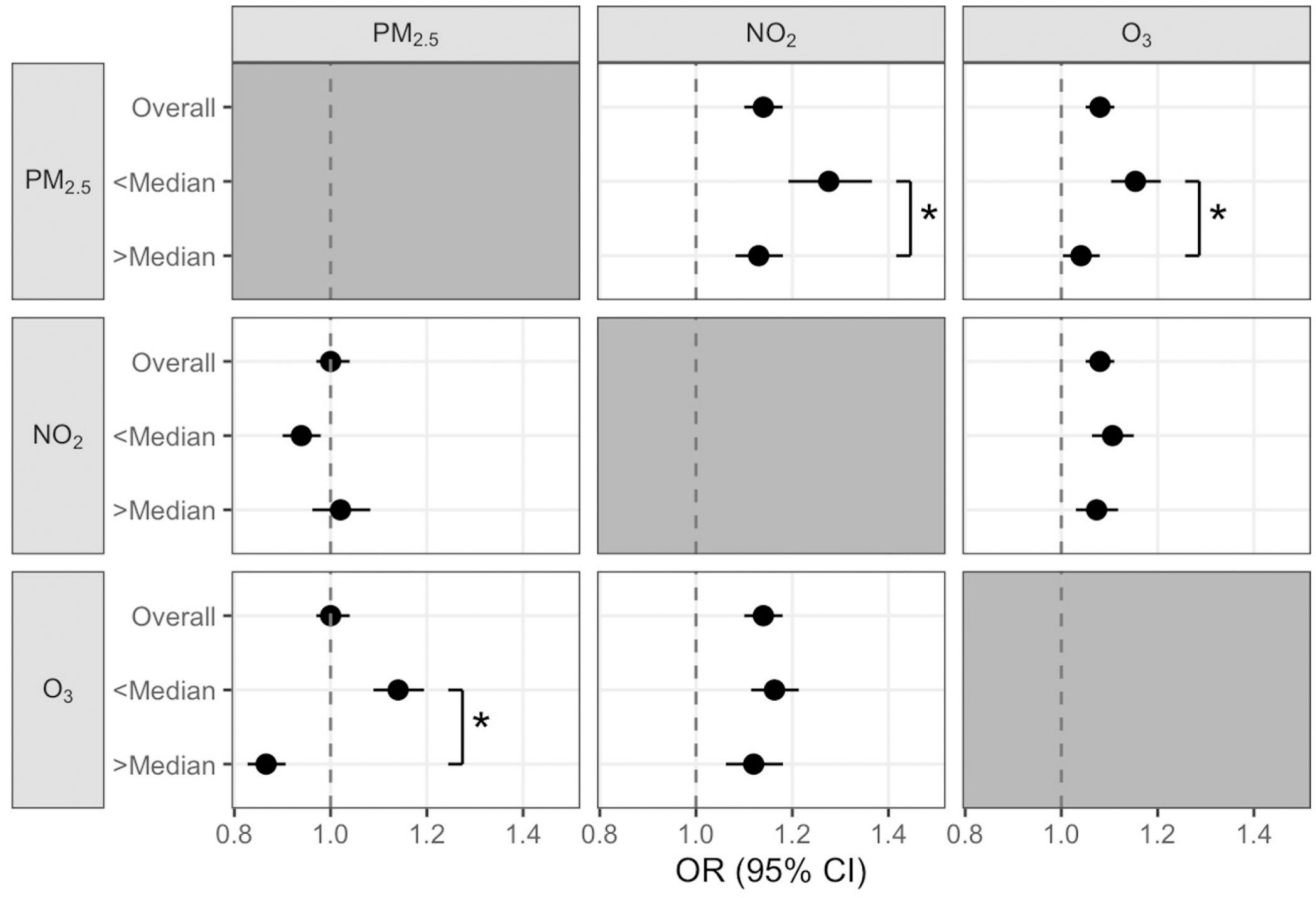
Two-way stratifications (below or above median) and interactions for prenatal exposures to particulate matter with a diameter 2.5μm or smaller (PM_2.5,_ in ug/m^3^), nitrogen dioxide (NO_2_, in ppb), ozone (O_3_, in ppb) and the risk of cerebral palsy. Asterisk (*) in the plot indicate the p-value for interaction between the two pollutants was below 0.05; the median levels of PM_2.5_ is 11.35 ug/m^3^, NO_2_ is 12.60 ppb, O_3_ is 37.54 ppb.

Per IQR increase in the maternal pregnancy-averaged exposure to NO_2_ (OR 1.14, 95% confidence interval (CI) 1.10–1.18) or O_3_ (OR 1.08, 95% CI 1.05–1.11) was associated with an increased odd of childhood CP. These associations were strengthened in multiple-pollutant models, and in exposure analyses of the first and third trimesters (Fig. 1, eTable 4). Further, stronger associations between NO_2_ or O_3_ and CP were also observed when the PM_2.5_ level was low (below median) (p-values for interactions <0.05) (Fig. 2, eTable 5). PM_2.5_ had a null association with CP overall, but a positive association emerged for PM_2.5_ when O_3_ was low (below median) (OR 1.14, 95% CI 1.09–1.19) (Fig. 2, eTable 5). In the chemical mixture analysis, the estimated joint effect of all three pollutants as a mixture was associated with 1.23 times higher odds for CP (95% CI 1.17–1.29, Fig. 1).

**Fig. 3.**
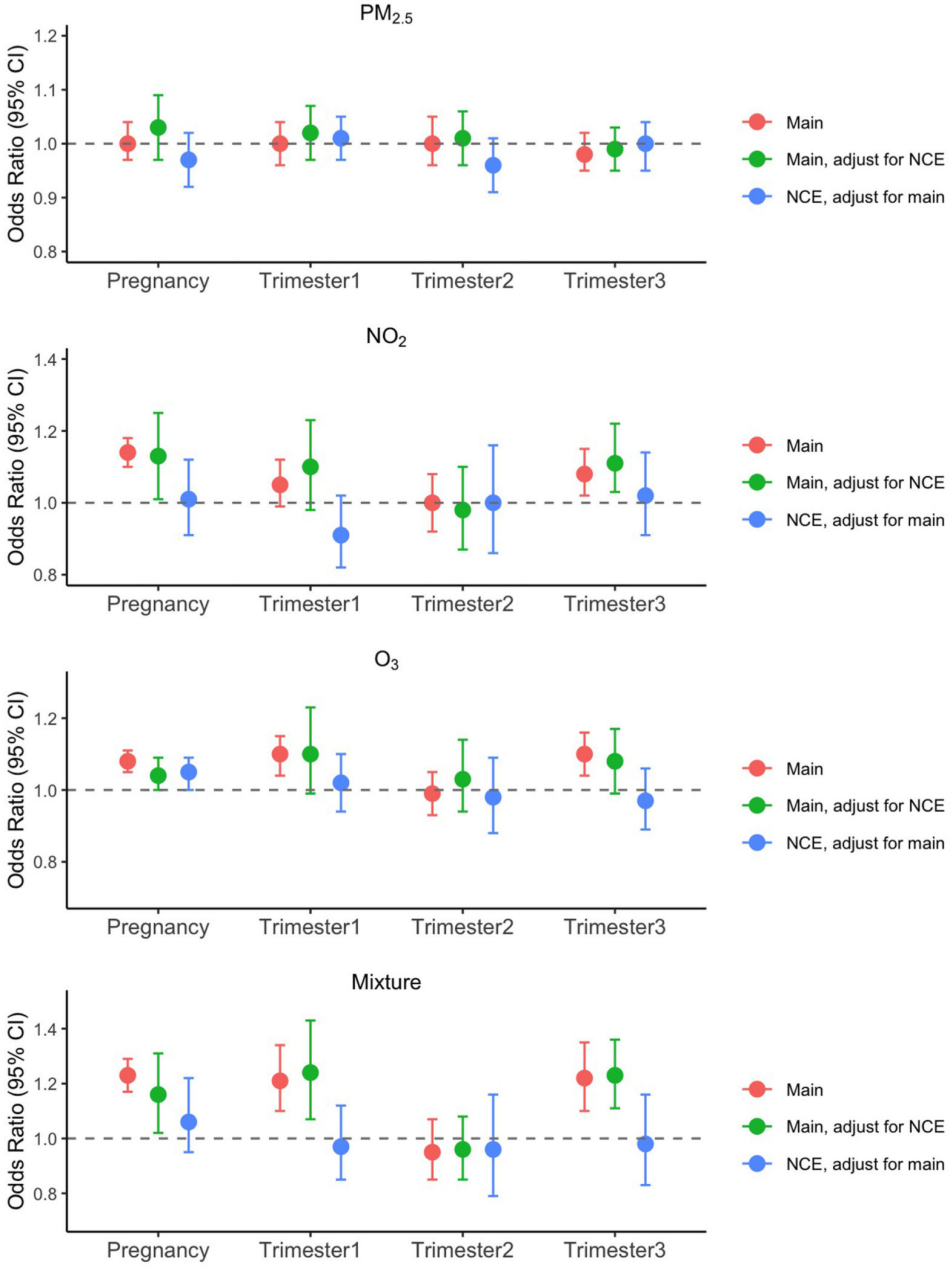
Associations between ambient particulate matter with a diameter 2.5μm or smaller (PM_2.5,_ in ug/m^3^), nitrogen dioxide (NO_2_, in ppb), ozone (O_3_, in ppb), and a chemical mixture during pregnancy and in the negative control period, with the risk of cerebral palsy. Estimated odds ratio (ORs) and 95% confidence intervals (CIs) of cerebral palsy for ambient air pollution at per interquartile range (IQR) increase, adjusted for year of birth, season of conception, maternal individual characteristics (age at delivery, race/ethnicity, education level, primary insurance type of prenatal care), zip-code level urban rural commuting code, and census-tract social vulnerability index (total score). Single pollution model was fitted, and the trimester-specific exposures were co-adjusted in the same model. Estimates represented in red dots were derived from models included the index exposures during pregnancy only. Estimates in green dots were the index exposures during pregnancy with co-adjustment for negative control exposure (NCE), and estimates in blue dots were NCE with co-adjustment for the index exposures during pregnancy.

The main findings remained largely unchanged in sensitivity analyses adjusting for additional confounding factors (eTable 6), using multiple imputation to replace missing covariates (eTable 7), assessing pregnancy exposure up to 32 gestational weeks (eTable 8), limiting to term births (eTable 9), or excluding CP cases with postnatal injury (eTable 10). Overall, no association was found between exposure at 36 months post-birth and CP, while the associations for the index exposures during pregnancy did not markedly change after co-adjusting the NCE variable (Fig. 3, eTable 11).

The associations for prenatal NO_2_ were stronger among spastic CP and dyskinetic CP, while the associations for prenatal O_3_ were stronger for dyskinetic CP and ataxic CP, which are rarer subtypes (Table 2). No apparent differences were found across the severity or the affected limbs of spastic CP, except PM_2.5_ was associated with spastic CP with mild to moderate severity (Table 2). The estimated mediating effects through preterm delivery, low birth weight, and small for gestational age were minimal, with the proportion mediated ranging from 1.5 % to 12.9% (eTable 12). There was no consistent effect modifications observed across child’s sex (eTable 13), neighborhood social vulnerability indexes, or maternal individual-level socio-economic indicators (eTable 14).

## 5. Discussion

Maternal prenatal exposures to residential ambient NO_2_ and O_3_ were associated with a higher risk of CP in the offspring, especially in the first and third trimesters. We observed potential interactions for the examined pollutants, as well as a mixture exposure effect from multiple pollutants. Additionally, NO_2_ or O_3_ may disproportionally affect certain CP subtypes. Negative control analyses did not indicate unmeasured confounding bias.

Two Canadian studies have recently reported that prenatal exposures to total PM_2.5_ (Zhang et al., 2024) and the SO^2^_4_^−^ component of PM_2.5_ (Ahmed et al., 2025) were associated with CP risk, while no effects were found for NO_2_ and O_3_ (Zhang et al., 2024). One possible explanation was that the spatial resolution for NO_2_ and O_3_ in our study was much finer than in previous studies (100 m vs. 10–21 km) (Zhang et al., 2024), which would substantially reduce measurement errors for these highly spatially-heterogeneous pollutants (Liu et al., 2020). Secondly, only term births were examined in previous Canadian studies (Zhang et al., 2024; Ahmed et al., 2025). Nevertheless, our findings did not change after excluding births with gestational age < 37 weeks in sensitivity analyses, suggesting the observed associations between NO_2_ and O_3_ with CP are not explained by the inclusion of both term and preterm births.

In California, NO_2_ is a well-recognized proxy for traffic-related air pollution (TRAPs) (Beckerman et al., 2008), and our smaller study in California has also found volatile organic compounds (VOCs) from exhaust and metals from brake and tire to be associated with CP risk (Zhuo et al., 2025). Other California studies have reported prenatal exposure to NO_2_ being associated with impaired psychomotor function in childhood (Shang et al., 2020) and other types of early-childhood neurodevelopmental disorders (Volk et al., 2021). California has a stricter air quality standard for NO_2_ than any other state in the US (30 vs. 53 ppb for annual average) (California Air Resources Board., 2025), and the NOx emission levels in California are lower than in many other states in the US (Environmental Protection Agency, 2023). Yet, our findings showed that even levels of ambient NO_2_ below the current standard of “no harmful health effects” could influence childhood CP risk; a 10–20% increase in odds were observed for CP when examining an inter-quartile range increase of NO_2_ from 9.74 ppb to 18.23 ppb in the population. Such an exposure related health burden could potentially be even greater in other states in the US with higher ambient NO_2_ levels.

Unlike NOx and PM_2.5_, O_3_ concentrations are predicted to increase in California (eFig. 3) (Zhao et al., 2024). Ground-level O_3_ is dominantly formed from complex photochemical reactions of primary precursors of NOx and VOCs under sunlight, inducing highest concentrations in or just downwind of large cities during sunny summer season (Council and Do, 2008). In California, the most heavily populated areas like southern California experienced the worst ozone levels in the US (Council and Do, 2008). Impaired motor function and motor developmental delays have been linked to O_3_ (Iglesias-Vázquez et al., 2022; Shu et al., 2024; Ha et al., 2019; Zou et al., 2021), as have several other major neurodevelopmental disorders (Chiu et al., 2023; Grineski et al., 2024; Zhou et al., 2023; Kaufman et al., 2019). In fact, the U.S. EPA has concluded that both long-term and short-term exposures to O_3_ are suggestively associated with central nervous system effects, but evidence is insufficient to establish causality (U.S. EPA, 2020).

Unlike previous Canadian studies reporting an association between the total PM_2.5,_ particularly the sulfate component, and CP risk, we did not find strong associations in California. One possible explanation is that the PM_2.5_ composition differs in the two study regions. PM_2.5_ represents a mixture of airborne particles and chemicals whose composition varies greatly across locations and seasons, influenced by meteorological conditions and emission sources. Previous Canadian studies reported an association between PM_2.5_, especially its sulfate component, and CP risk, attributing this to the historically recorded high sulfate emissions in the Ontario region (Ahmed et al., 2025). We did not find such associations in California where sulfate contributions to PM_2.5_ are negligible, instead it has a large road traffic related components (Hasheminassab et al., 2014). We found an increased risk of CP with PM_2.5_ when O_3_ levels are low. In California, the negative correlations between PM_2.5_ and O_3_ are more pronounced in the winter season (November - February) (Council and Do, 2008), suggesting that PM_2.5_ generated from traffic in winter when O_3_ exposure is low might induce stronger developmental neurotoxicity. On the contrary, there was a counterintuitive inverse association between CP and PM_2.5_ under high O_3_ exposure. One possible reason could be that traffic components of PM_2.5_ may be better captured by the NO_2_ than PM_2.5_ LUR measures, as traffic contributions to PM_2.5_ are likely low where ozone levels are high. Secondly, PM_2.5_ from photochemical smog or wildfires in late summer may have more toxic components that induce fetal loss, leading to live-birth bias and a counter-intuitively lower risk of CP. Further, behavioral changes such as avoiding being outdoors when there is heavy smoke or extreme heat during the hot season might also contribute. Additionally, other uncontrolled factors might interact with PM_2.5_ or O_3_, and collider bias may occur in stratified analyses when uncontrolled predictors or precursors of O_3_ influence CP risk. Overall, PM_2.5_ may show heterogeneous health impacts depending on the mixture particle composition and sources, and which PM_2.5_ measure or modelling approaches best capture the toxic components. Future research that incorporates source-specific PM_2.5_ or pregnancy loss data are recommended to better explain these findings.

Biological plausibility of ambient air pollution’s effect on CP development includes neuroinflammation, either indirectly via maternal systemic inflammation or directly via transplacental and fetal central nervous system exposure to airborne particles (Block et al., 2012). Additionally, the oxidative stress from air pollution could increase glial activations to release inflammatory mediators that cause white matter injury and neurons damages (Juacy Rodrigues Costa-de-Santana et al., 2023; Boda et al., 2020). Other pathways such as elevated DNA adducts or abnormal release of dopamine in the cerebral cortex under prenatal exposures to particles and NO_2_ (Thomson, 2019; Levesque et al., 2011), or the release of stress hormones from activated hypothalamic-pituitary-adrenal (HPA) axis under exposures to O_3_ (Thomson, 2019), may also play important role in neuromotor function. Although the most sensitive window of prenatal exposures for CP development remains unclear, we observed a stronger association in the first trimester that was also suggested by the previous Canadian study that reported on the importance of exposure in weeks 4–9 of gestation (Ahmed et al., 2025). Additionally, exposures in the first trimester have also been implicated in previous studies on CP focused on other environmental factors like pesticides, ambient temperature, and antibiotic medication (Liew et al., 2020; Zhuo et al., 2026; Miller et al., 2013). Finally, air pollution could induce placental damage or disrupt blood flow to the fetus, triggering adverse birth outcomes and neurological effects that are more relevant in late pregnancy (Fussell et al., 2024), which however, was indicated to be minimal in our mediation analyses.

## 6. Strengths and limitations

Our study makes important contributions. We employed a newly developed high-resolution exposure model that minimizes exposure misclassification arising from spatial heterogeneity of air pollutants. Our study is nested within the largest population-based cohort on CP, and the registry-based linkage design eliminated the risk of recall bias and self-selection bias. Additionally, we evaluated unmeasured confounding using NCE analyses. Finally, our large case sample enables investigation of different CP subtypes that emerged from impairments in different regions of the developing brain, which may help to better understand the complex etiology of CP and timing of exposure.

Nevertheless, there are also limitations. First, our reliance on maternal residential address at delivery for prenatal exposure might have introduced exposure measurement error if there were residential mobilities. The misclassification is likely non-differential, and the potential bias magnitude should be small given that most mothers tend to move locally (Bell and Belanger, 2012). Second, the validity of the NCE analyses relies on an assumption that the unmeasured confounding structure is similar between the index and the NCE exposure periods. We estimated the NCE using the same residential address at delivery as used for the index exposure primarily to address spatial confounding. The goal of our NCE is not to study the actual postnatal exposure, since the children could have moved from their birth addresses, but to examine whether a predefined exposure measure that does not causally affect the outcome suggests uncontrolled confounding. Nevertheless, this NCE does not rule out other time-varying confounding factors. Third, less accuracy of the predicted total mass of PM_2.5_ from the LUR model might limit our ability to detect health effects, especially in places where fewer monitoring sites were available. Nevertheless, the LUR employed is one of the most sophisticated and up to date exposure models for California. Fourth, DDS may not capture mild CP cases that are in less need of services, which can contribute to outcome misclassification bias. However, the number of CP being misclassified in the control group is likely to be very small given the rarity of CP prevalence. While CP is not considered a heritable condition, future research that incorporates genetic data may shed light on the joint actions of genetic susceptibility and air pollution exposures on CP development. Finally, restriction to live births in our analyses might induce live birth bias with a potential downward bias when the exposure affects pregnancy or fetal losses and in those who are at greater risk for CP (Leung et al., 2021).

## 7. Conclusion

Our 16-year statewide birth cohort from California shows that prenatal exposure to ambient air pollution is associated with an increased risk of CP. Further studies are needed to confirm our findings and to elucidate the underlying biological mechanisms.

## Supplementary Material

Supplement materials

## Figures and Tables

**Table 1:** Characteristics of the cerebral palsy cases and controls in California, 2000–2015.

Characteristics		N (%) ^1^	
		
		Cerebral palsy (CP) (N= 9,343)	Non-CP controls (N=1,560,423)

**Main covariates**			
Maternal age at delivery			
	<=18	570 (6.1)	78,378 (5.0)
	19–25	2,736 (29.3)	462,249 (29.6)
	26–30	2,327 (24.9)	422,552 (27.1)
	31–35	2,131 (22.8)	377,393 (24.2)
	>35	1,576 (16.9)	219,824 (14.1)
	Unknown	3 (<0.1)	27 (<0.1)
Maternal race and ethnicity			
	African	743 (8.0)	89,482 (5.7)
	American or		
	Black		
	Asian	823 (8.8)	199,378 (12.8)
	Hispanic or	4,956 (53.0)	776,917 (49.8)
	Latinx of any race		
	Non-Hispanic	2,592 (27.7)	456,183 (29.2)
	White		
	Others ^2^	90 (1.0)	15,195 (1.0)
	Unknown	139 (1.5)	23,268 (1.5)
Maternal education level			
	<12th grade	2,591 (27.7)	381,080 (24.4)
	High school or diploma	4,688 (50.2)	742,633 (47.6)
	College and above	1,815 (19.4)	390,011 (25.0)
	Unknown	249 (2.7)	46,699 (3.0)
Payment source for prenatal care			
	Government ^3^	4,679 (50.1)	730,923 (46.8)
	Private	4,277 (45.8)	768,499 (49.2)
	Others	365 (3.9)	58,337 (3.7)
	Unknow/unreported	22 (0.2)	2,664 (0.2)
Season of conception			
	Winter	2,199 (23.5)	374,016 (24.0)
	Spring	2,334 (25.0)	379,895 (24.3)
	Summer	2,288 (24.5)	384,874 (24.7)
	Fall	2,522 (27.0)	421,638 (27.0)
Birth year			
	2000–2003	2,808 (30.1)	384,917 (24.7)
	2004–2007	2,456 (26.3)	397,554 (25.5)
	2008–2011	2,256 (24.1)	395,043 (25.3)
	2012–2015	1,823 (19.5)	382,909 (24.5)
Sex of children			
	Male	5,208 (55.7)	799,011 (51.2)
	Female	4,135 (44.3)	761,412 (48.8)
Social vulnerability index, median (SD) ^4^		0.67 (0.28)	0.62 (0.28)
Rural urban commuting code			
	Metropolitan	8,906 (95.3)	1,478,620 (94.8)
	Micropolitan	318 (3.4)	57,420 (3.7)
	Small town/rural	118 (1.3)	24,272 (1.5)
	Unknown	1 (<0.1)	111 (<0.1)
**Mediators**			
Preterm birth (< 37 complete weeks)		3,194 (34.2)	158,938 (10.2)
Very preterm birth (< 32 complete weeks)		1,763 (18.9)	22,899 (1.5)
Low birth weight (<2500 gram)		3,249 (34.8)	101,872 (6.5)
Small for gestational age (< 10^th^ percentile at each gestational age)		1,978 (21.2)	152,932 (9.8)

1 May does not add to 100% due to rounding.

2 Other race and ethnicity collapsed Pacific Islander, American Indian, Eskimo, Aleut, and other unspecified owing to small sample size.

3 Government insurance types included Medical and other government programs at Federal, State, Local level.

4 Census tract level vulnerability index ranging 0 to 1, with higher score indicates higher vulnerability.

**Table 2:** Associations between ambient air pollution and the risk of cerebral palsy, stratified by cerebral palsy subtypes.

	Sample size Case/Control	PM_2.5_ OR (95% CI)	NO_2_ OR (95% CI)	O_3_ OR (95% CI)

Cerebral palsy subtypes				
Spastic	6,631/1,560,423	1.03 (0.99, 1.07)	1.20 (1.15, 1.25)	1.11 (1.07, 1.14)
Ataxic	510/1,560,423	0.87 (0.76, 0.99)	0.93 (0.81, 1.07)	1.20 (1.09, 1.32)
Dyskinetic	253/1,560,423	1.01 (0.97, 1.05)	1.13 (1.08, 1.18)	1.34 (1.14, 1.61)
Mixed	1,949/1,560,423	0.98 (0.91, 1.05)	1.05 (0.98, 1.13)	0.94 (0.88, 1.01)
Severity of motor dysfunction among spastic CP				
Mild and moderate	4,060/1,560,423	1.07 (1.02, 1.12)	1.18 (1.12, 1.24)	1.13 (1.08, 1.18)
Severe	2,571/1,560,423	0.97 (0.91, 1.03)	1.22 (1.15, 1.30)	1.07 (1.02, 1.13)
Limb involvement of spastic CP				
Unilateral	1,873/1,560,423	0.98 (0.90, 1.07)	1.16 (1.06, 1.27)	1.11 (1.03, 1.20)
Bilateral	4,758/1,560,423	1.03 (1.00, 1.09)	1.18 (1.13, 1.24)	1.16 (1.12, 1.20)

Estimated odds ratio (ORs) and 95% confidence intervals (CIs) of cerebral palsy for ambient air pollution at per interquartile range (IQR) increase, adjusted for year of birth, season of conception, maternal individual characteristics (age at delivery, race/ethnicity, education level, primary insurance type of prenatal care), zip-code level urban rural commuting code, and census-tract social vulnerability index (total score).

## Data Availability

The data that support findings from this study are from the California Department of Public Health and Department of Developmental Services. The authors have no rights to share the data due to restrictions on human subject protocol and data sharing policies set by the State of California. Interested researchers can submit applications to the California Health and Human Services Agency Committee for the Protection of Human Subjects. Codes for all statistical analysis are available on the group Github Page (https://github.com/Liew-Lab/Cerebral-Palsy-Study-in-California).

## Appendix A. Supplementary data

Supplementary data to this article can be found online at https://doi.org/10.1016/j.envint.2026.110201.
