# Supplementary material for: Ambient air pollution during pregnancy and offspring cerebral palsy
Source: Environ Int. Author manuscript; Available in PMC 2026 Jun 27. (PMC13309808; doi:10.1016/j.envint.2026.110201)
Supplement: Supplement materials [file NIHMS2180308-supplement-Supplement_materials.docx]

**Supplementary Materials**

**Ambient air pollution during pregnancy and offspring cerebral palsy**

Haoran Zhuo, PhD^1,2^; Beate Ritz, MD, PhD^3,4^; Jason G. Su, PhD^5^; Roch A. Nianogo, MD, PhD^3^; Joshua Warren, PhD^6^; Zeyan Liew, PhD^1,2^

**Supplementary Tables**

**eTable 1**. Characteristics of additional covariates between cerebral palsy cases and controls in California, 2000-2015

**eTable 2**. Ambient air pollutants distribution among study population, 2000-2015.

**eTable 3**. Pearson's correlation coefficient among ambient air pollutants.

**eTable 4**. Associations between ambient particulate matter with a diameter 2.5μm or smaller (PM_2.5_, in ug/m3), nitrogen dioxide (NO_2_, in ppb), ozone (O_3_, in ppb) during pregnancy and the risk of cerebral palsy (results presented in Figure 1).

**eTable 5**. Two-way stratifications (below or above median) and interactions for prenatal exposures to particulate matter with a diameter 2.5μm or smaller (PM_2.5_, in ug/m3), nitrogen dioxide (NO_2_, in ppb), ozone (O_3_, in ppb) and the risk of cerebral palsy (results presented in Figure 2).

**eTable 6**. Associations between ambient particulate matter with a diameter 2.5μm or smaller (PM_2.5_, in ug/m3), nitrogen dioxide (NO_2_, in ppb), ozone (O_3_, in ppb) during pregnancy and the risk of cerebral palsy, adjusted for additional covariates.

**eTable 7**. Associations between ambient particulate matter with a diameter 2.5μm or smaller (PM_2.5_, in ug/m3), nitrogen dioxide (NO_2_, in ppb), ozone (O_3_, in ppb) during pregnancy and the risk of cerebral palsy, with multiple imputation on missing covariates.

**eTable 8**. Associations between ambient particulate matter with a diameter 2.5μm or smaller (PM_2.5_, in ug/m3), nitrogen dioxide (NO_2_, in ppb), ozone (O_3_, in ppb) during pregnancy and the risk of cerebral palsy, limited to 32 gestational weeks.

**eTable 9**. Associations between ambient particulate matter with a diameter 2.5μm or smaller (PM_2.5_, in ug/m3), nitrogen dioxide (NO_2_, in ppb), ozone (O_3_, in ppb) during pregnancy and the risk of cerebral palsy, limited to term births (≥ 37 gestational weeks).

**eTable 10**. Associations between ambient particulate matter with a diameter 2.5μm or smaller (PM_2.5_, in ug/m3), nitrogen dioxide (NO_2_, in ppb), ozone (O_3_, in ppb) during pregnancy and the risk of cerebral palsy, excluded accident-related CP cases.

**eTable 11**. Associations between ambient particulate matter with a diameter 2.5μm or smaller (PM_2.5,_ in ug/m^3^), nitrogen dioxide (NO_2_, in ppb), ozone (O_3_, in ppb), and a chemical mixture during pregnancy and in the negative control period, with the risk of cerebral palsy (results presented in Figure 3).

**eTable 12**. Mediation analysis of adverse birth outcomes in the associations between ambient air pollution and cerebral palsy.

**eTable 13**. Associations between ambient air pollution and the risk of cerebral palsy, stratified by child’s sex.

**eTable 14**. Associations between ambient air pollution and the risk of cerebral palsy, stratified analyses by maternal neighborhood and individual socioeconomic status.

**Supplementary Figures**

**eFigure 1**. Directed acyclic graph for the confounding structure.

**eFigure 2**. Flowchart of the study population.

**eFigure 3**. Median levels of prenatal exposures to particulate matter with a diameter 2.5μm or smaller (PM_2.5_), nitrogen dioxide (NO_2_), and ozone (O_3_) across years.

**eTable 1. Characteristics of additional covariates between cerebral palsy cases and controls in California, 2000-2015**

| **Characteristics** | | **N (%) ^1^** | | |  |  |
| --- | --- | --- | --- | --- | --- | --- |
|  |  | **Cerebral palsy (CP)**  **(N= 9,343)** | **Non-CP controls**  **(N=1,560,423)** |  |  |  |
| **Additional covariates in sensitivity analyses** | |  |  |  |  |  |
| Parity | |  |  |  |  |  |
|  | Primipara | 3,781 (40.5) | 608,317 (39.0) |  |  |  |
|  | Para 2 | 2,655 (28.4) | 494,088 (31.7) |  |  |  |
|  | Para 3 or more | 2,904 (31.1) | 457,331 (29.3) |  |  |  |
|  | Uknown | 3 (<0.1) | 687 (<0.1) |  |  |  |
| Maternal pre-pregnancy body mass index (BMI) ^2^ | |  |  |  |  |  |
|  | <18.5 | 156 (3.3) | 33,448 (3.8) |  |  |  |
|  | 18.5 to <25.0 | 1,847 (39.3) | 407,458 (46.1) |  |  |  |
|  | 25.0 to <30.0 | 1,204 (25.6) | 213,594 (24.2) |  |  |  |
|  | ≥ 30.0 | 1,165 (24.8) | 174,553 (19.7) |  |  |  |
|  | Unknown | 330 (7.0) | 54,957 (6.2) |  |  |  |
| Cigarette smoking before pregnancy ^2^ | | 199 (4.2) | 27,281 (3.1) |  |  |  |
| Cigarette smoking during pregnancy ^2^ | | 154 (3.3) | 18,576 (2.1) |  |  |  |
| Receipt of Women, infants, and children (WIC) food supplements ^2^ | | 2,725 (58.0) | 457,498 (51.8) |  |  |  |
| Ambient temperature during pregnancy (°C), median (SD) | | 17.85 (2.63) | 17.64 (2.38) |  |  |  |
| ^1^ May does not add to 100% due to rounding | | | | | |  |
| ^2^ Only available in birth years of 2007-2015. | | | | | |  |

| **eTable 2. Ambient air pollutants distribution among study population, 2000-2015.** | | | | | | | | | | | | |
| --- | --- | --- | --- | --- | --- | --- | --- | --- | --- | --- | --- | --- |
|  |  | Non-CP controls, median (IQR) | | |  | CP cases, median (IQR) | | | | | | |
|  | N | PM_2.5_ (ug/m^3^) | NO_2_ (ppb) | O_3_ (ppb) | N | PM_2.5_ (ug/m^3^) | | NO_2_ (ppb) | | O_3_ (ppb) | | |
| **Prenatal exposure** | |  |  |  |  |  | |  | |  | | |
| Pregnancy | 1,560,423 | 11.35 (2.84) | 12.60 (8.49) | 37.54 (3.77) | 9,343 | 11.58 (2.75) | 13.70 (9.04) | | 37.59 (3.99) | | |  |
| Trimester 1 | 1,560,423 | 11.36 (3.13) | 12.85 (8.77) | 37.19 (7.98) | 9,343 | 11.61 (3.01) | 13.85 (9.20) | | 37.34 (7.79) | | |  |
| Trimester 2 | 1,560,423 | 11.27 (3.16) | 12.58 (8.68) | 37.63 (8.06) | 9,343 | 11.50 (3.06) | 13.57 (9.27) | | 37.73 (7.92) | | |  |
| Trimester 3 | 1,551,779 | 11.19 (3.16) | 12.41 (8.54) | 37.67 (8.52) | 8,315 | 11.41 (3.04) | 13.40 (9.14) | | 37.72 (8.66) | | |  |
| **Negative control exposure** | | |  |  |  |  |  | |  | |  |  |
| Pregnancy | 1,560,423 | 10.63 (2.77) | 11.23 (7.11) | 37.66 (3.66) | 9,343 | 10.82 (2.71) | 12.17 (7.67) | | 37.78 (3.88) | |  |  |
| Trimester 1 | 1,560,423 | 10.65 (3.02) | 11.28 (7.78) | 37.64 (7.93) | 9,343 | 10.85 (2.87) | 12.18 (8.41) | | 37.80 (7.96) | |  |  |
| Trimester 2 | 1,560,423 | 10.60 (3.04) | 11.20 (7.79) | 37.45 (7.92) | 9,343 | 10.79 (2.94) | 12.17 (8.38) | | 37.67 (7.81) | |  |  |
| Trimester 3 | 1,551,796 | 10.50 (3.10) | 10.96 (7.80) | 37.87 (8.49) | 8,315 | 10.69 (2.98) | 11.80 (8.45) | | 37.87 (8.55) | |  |  |

| **eTable 3. Pearson’s correlation coefficients among ambient air pollutants** | | | |
| --- | --- | --- | --- |
|  | NO_2_ & O_3_ | PM_2.5_  & NO_2_ | PM_2.5_ & O_3_ |
| Pregnancy | -0.15 | 0.43 | 0.17 |
| Trimester 1 | -0.37 | 0.40 | -0.15 |
| Trimester 2 | -0.37 | 0.42 | -0.13 |
| Trimester 3 | -0.38 | 0.41 | -0.12 |
|  |  |  |  |
|  | NO_2_ | PM_2.5_ | O_3_ |
| Trim1 & Trim2 | 0.63 | 0.58 | 0.18 |
| Trim1 & Trim3 | 0.28 | 0.43 | -0.50 |
| Trim2 & Trim3 | 0.62 | 0.58 | 0.16 |

|  | **eTable 4. Associations between ambient particulate matter with a diameter 2.5μm or smaller (PM_2.5_, in ug/m3), nitrogen dioxide (NO_2_, in ppb), ozone (O_3_, in ppb) during pregnancy and the risk of cerebral palsy (results presented in Figure 1).** | | | |
| --- | --- | --- | --- | --- |
|  | | **Single pollutant model**  **OR (95% CI)** | **Multiple pollutants model**  **OR (95% CI)** | **Mixture model**  **OR (95% CI)** |
| **PM_2.5_** | |  |  |  |
| Pregnancy | | 1.00 (0.97, 1.04) | 0.91 (0.87, 0.94) | Pregnancy: 1.23 (1.17, 1.29)  Trimester 1: 1.21 (1.10, 1.34)  Trimester 2: 0.95 (0.85, 1.07)  Trimester 3: 1.22 (1.10, 1.35) |
| Trimester 1 | | 1.00 (0.96, 1.04) | 0.96 (0.91, 1.00) |  |
| Trimester 2 | | 1.00 (0.96, 1.05) | 0.96 (0.91, 1.01) |  |
| Trimester 3 | | 0.98 (0.95, 1.02) | 0.95 (0.90, 0.99) |  |
| **NO_2_** | |  |  |  |
| Pregnancy | | 1.14 (1.10, 1.18) | 1.21 (1.17, 1.26) |  |
| Trimester 1 | | 1.05 (0.99, 1.12) | 1.09 (1.02, 1.15) |  |
| Trimester 2 | | 1.00 (0.92, 1.08) | 1.01 (0.93, 1.09) |  |
| Trimester 3 | | 1.08 (1.02, 1.15) | 1.12 (1.05, 1.19) |  |
| **O_3_** | |  |  |  |
| Pregnancy | | 1.08 (1.05, 1.11) | 1.12 (1.09, 1.16) |  |
| Trimester 1 | | 1.10 (1.04, 1.15) | 1.17 (1.10, 1.23) |  |
| Trimester 2 | | 0.99 (0.93, 1.05) | 0.99 (0.93, 1.05) |  |
| Trimester 3 | | 1.10 (1.04, 1.16) | 1.16 (1.09, 1.23) |  |

Estimated odds ratio (ORs) and 95% confidence intervals (CIs) of cerebral palsy for ambient air pollution at per interquartile range (IQR) increase, adjusted for year of birth, season of conception, maternal individual characteristics (age at delivery, race/ethnicity, education level, primary insurance type of prenatal care), zip-code level rural-urban commuting code, and census-tract social vulnerability index (total score).

Trimester-specific exposures were co-adjusted in the same model. Multiple pollutant model was modelled by co-adjusting all three pollutants in the same model. Mixture model was fitted use quantile g-computation method, treating all three pollutants as a chemical mixture in each exposure window.

**eTable 5. Two-way stratifications (below or above median) and interactions for prenatal exposures to particulate matter with a diameter 2.5μm or smaller (PM_2.5_, in ug/m3), nitrogen dioxide (NO_2_, in ppb), ozone (O_3_, in ppb) and the risk of cerebral palsy (results presented in Figure 2).**

|  |  | **PM_2.5_**  **OR (95% CI)** | **NO_2_**  **OR (95% CI)** | **O_3_**  **OR (95% CI)** |
| --- | --- | --- | --- | --- |
| **PM_2.5_** | |  |  |  |
|  | < median (11.35 ug/m^3^) | -- | 1.28 (1.19, 1.37) | 1.15 (1.10, 1.21) |
|  | > median | -- | 1.13 (1.08, 1.18) | 1.04 (1.00, 1.08) |
|  | P-value for interaction | -- | 0.006 | <0.0001 |
| **NO_2_** | | | | |
|  | < median (12.60 ppb) | 0.94 (0.90, 0.98) | -- | 1.11 (1.06, 1.15) |
|  | > median | 1.02 (0.96, 1.08) | -- | 1.07 (1.03, 1.22) |
|  | P-value for interaction | 0.309 | -- | 0.293 |
| **O_3_** | | | | |
|  | < median (37.54 ppb) | 1.14 (1.09, 1.19) | 1.16 (1.11, 1.21) | -- |
|  | > median | 0.87 (0.83, 0.91) | 1.12 (1.06, 1.18) | -- |
|  | P-value for interaction | <0.0001 | 0.161 | -- |

Estimated odds ratio (ORs) and 95% confidence intervals (CIs) of cerebral palsy for ambient air pollution at per interquartile range (IQR) increase, adjusted for year of birth, season of conception, maternal individual characteristics (age at delivery, race/ethnicity, education level, primary insurance type of prenatal care), zip-code level rural-urban commuting code and census-tract social vulnerability index (total score). P-value for interaction was estimated by adding an interaction term between the main pollutant and the stratified pollutant in the model.

| **eTable 6. Associations between ambient particulate matter with a diameter 2.5μm or smaller (PM_2.5_, in ug/m3), nitrogen dioxide (NO_2_, in ppb), ozone (O_3_, in ppb) during pregnancy and the risk of cerebral palsy, adjusted for additional covariates** | | | | |
| --- | --- | --- | --- | --- |
|  | **Main single-pollutant model**  **OR (95% CI)** | **Main single-pollutant model, additional covariates**  **OR (95% CI)** | **Main multi-pollutant model**  **OR (95% CI)** | **Main multi-pollutant model,**  **additional covariates**  **OR (95% CI)** |
| **PM_2.5_** |  |  |  |  |
| Pregnancy | 1.00 (0.97, 1.04) | 0.97 (0.93, 1.02) | 0.91 (0.87, 0.94) | 0.87 (0.83, 0.92) |
| Trimester 1 | 1.00 (0.96, 1.04) | 1.00 (0.95, 1.05) | 0.96 (0.91, 1.00) | 0.95 (0.90, 1.01) |
| Trimester 2 | 1.00 (0.96, 1.05) | 0.98 (0.92, 1.03) | 0.96 (0.91, 1.01) | 0.93 (0.87, 0.99) |
| Trimester 3 | 0.98 (0.95, 1.02) | 0.98 (0.94, 1.03) | 0.95 (0.90, 0.99) | 0.95 (0.90, 1.01) |
| **NO_2_** |  |  |  |  |
| Pregnancy | 1.14 (1.10, 1.18) | 1.18 (1.13, 1.24) | 1.21 (1.17, 1.26) | 1.24 (1.16, 1.31) |
| Trimester 1 | 1.05 (0.99, 1.12) | 1.10 (1.02, 1.18) | 1.09 (1.02, 1.15) | 1.12 (1.04, 1.20) |
| Trimester 2 | 1.00 (0.92, 1.08) | 0.99 (0.90, 1.09) | 1.01 (0.93, 1.09) | 1.00 (0.91, 1.10) |
| Trimester 3 | 1.08 (1.02, 1.15) | 1.12 (1.04, 1.20) | 1.12 (1.05, 1.19) | 1.14 (1.05, 1.23) |
| **O_3_** |  |  |  |  |
| Pregnancy | 1.08 (1.05, 1.11) | 1.09 (1.05, 1.14) | 1.12 (1.09, 1.16) | 1.12 (1.05, 1.19) |
| Trimester 1 | 1.10 (1.04, 1.15) | 1.07 (1.00, 1.15) | 1.17 (1.10, 1.23) | 1.13 (1.03, 1.23) |
| Trimester 2 | 0.99 (0.93, 1.05) | 0.99 (0.91, 1.07) | 0.99 (0.93, 1.05) | 0.96 (0.87, 1.06) |
| Trimester 3 | 1.10 (1.04, 1.16) | 1.09 (1.01, 1.18) | 1.16 (1.09, 1.23) | 1.17 (1.07, 1.27) |

Main models were fitted on birth years of 2000-2015, models with additional covariates were fitted on birth years of 2007-2015 due to the data availability. Estimated odds ratio (ORs) and 95% confidence intervals (CIs) of cerebral palsy for ambient air pollution at per interquartile range (IQR) increase, adjusted for year of birth, season of conception, maternal individual characteristics (age at delivery, race/ethnicity, education level, primary insurance type of prenatal care), census-tract social vulnerability index (total score), with additional adjustment for maternal pre-pregnancy body mass index, cigarette smoking before and during pregnancy, the receipt for Special Supplemental Nutrition Program for Women, Infants, and Children (WIC) benefits, and the ambient temperature over pregnancy.

Trimester-specific exposures were co-adjusted in the same model. Single-pollutant model analyzed each pollutant separately, and multiple-pollutant model was modelled by co-adjusting all three pollutants in the same model.

| **eTable 7. Associations between ambient particulate matter with a diameter 2.5μm or smaller (PM_2.5_, in ug/m3), nitrogen dioxide (NO_2_, in ppb), ozone (O_3_, in ppb) during pregnancy and the risk of cerebral palsy, using multiple imputation to replace missing covariates.** | | |
| --- | --- | --- |
|  | **Single pollutant model**  **OR (95% CI)** | **Multiple pollutants model**  **OR (95% CI)** |
| **PM_2.5_** |  |  |
| Pregnancy | 1.00 (0.97, 1.04) | 0.90 (0.87, 0.94) |
| Trimester 1 | 1.00 (0.96, 1.04) | 0.96 (0.91, 1.00) |
| Trimester 2 | 1.00 (0.96, 1.05) | 0.96 (0.91, 1.01) |
| Trimester 3 | 0.98 (0.95, 1.02) | 0.95 (0.90, 0.99) |
| **NO_2_** |  |  |
| Pregnancy | 1.14 (1.10, 1.18) | 1.21 (1.17, 1.26) |
| Trimester 1 | 1.05 (0.99, 1.12) | 1.09 (1.02, 1.15) |
| Trimester 2 | 1.00 (0.92, 1.08) | 1.01 (0.93, 1.09) |
| Trimester 3 | 1.08 (1.02, 1.15) | 1.12 (1.05, 1.19) |
| **O_3_** |  |  |
| Pregnancy | 1.08 (1.05, 1.11) | 1.12 (1.09, 1.16) |
| Trimester 1 | 1.10 (1.04, 1.15) | 1.16 (1.10, 1.23) |
| Trimester 2 | 0.99 (0.93, 1.05) | 0.99 (0.93, 1.05) |
| Trimester 3 | 1.10 (1.04, 1.16) | 1.16 (1.09, 1.23) |

Estimated odds ratio (ORs) and 95% confidence intervals (CIs) of cerebral palsy for ambient air pollution at per interquartile range (IQR) increase, adjusted for year of birth, season of conception, maternal individual characteristics (age at delivery, race/ethnicity, education level, primary insurance type of prenatal care), zip-code level rural-urban commuting code and census-tract social vulnerability index (total score).

Trimester-specific exposures were co-adjusted in the same model. Single-pollutant model analyzed each pollutant separately, and multiple-pollutant model was modelled by co-adjusting all three pollutants in the same model.

| **eTable 8. Associations between ambient particulate matter with a diameter 2.5μm or smaller (PM_2.5_, in ug/m3), nitrogen dioxide (NO_2_, in ppb), ozone (O_3_, in ppb) pregnancy and the risk of cerebral palsy, prenatal exposure window limited to 32 weeks of gestation.** | | |
| --- | --- | --- |
|  | **Single pollutant model**  **OR (95% CI)** | **Multiple pollutants model**  **OR (95% CI)** |
| **PM_2.5_** |  |  |
| Pregnancy | 1.00 (0.97, 1.03) | 0.92 (0.89, 0.96) |
| Trimester 1 | 1.00 (0.96, 1.04) | 0.96 (0.92, 1.01) |
| Trimester 2 | 1.01 (0.96, 1.06) | 0.96 (0.91, 1.02) |
| Trimester 3 | 0.98 (0.94, 1.02) | 0.95 (0.91, 0.99) |
| **NO_2_** |  |  |
| Pregnancy | 1.13 (1.09, 1.17) | 1.18 (1.14, 1.23) |
| Trimester 1 | 1.07 (1.00, 1.14) | 1.09 (1.03, 1.17) |
| Trimester 2 | 0.99 (0.90, 1.10) | 0.99 (0.89, 1.10) |
| Trimester 3 | 1.06 (0.99, 1.14) | 1.11 (1.03, 1.20) |
| **O_3_** |  |  |
| Pregnancy | 1.08 (1.05, 1.12) | 1.12 (1.08, 1.16) |
| Trimester 1 | 1.11 (1.05, 1.17) | 1.18 (1.11, 1.25) |
| Trimester 2 | 0.97 (0.91, 1.04) | 0.97 (0.90, 1.04) |
| Trimester 3 | 1.09 (1.03, 1.16) | 1.14 (1.06, 1.21) |

Estimated odds ratio (ORs) and 95% confidence intervals (CIs) of cerebral palsy for ambient air pollution at per interquartile range (IQR) increase, adjusted for year of birth, season of conception, maternal individual characteristics (age at delivery, race/ethnicity, education level, primary insurance type of prenatal care), zip-code rural-urban commuting code, and census-tract social vulnerability index (total score).

Trimester-specific exposures were co-adjusted in the same model. Single-pollutant model analyzed each pollutant separately, and multiple-pollutant model was modelled by co-adjusting all three pollutants in the same model.

| **eTable 9. Associations between ambient particulate matter with a diameter 2.5μm or smaller (PM_2.5_, in ug/m3), nitrogen dioxide (NO_2_, in ppb), ozone (O_3_, in ppb) during pregnancy and the risk of cerebral palsy, term birth (gestational weeks≥37) only.** | | |
| --- | --- | --- |
|  | **Single pollutant model**  **OR (95% CI)** | **Multiple pollutants model**  **OR (95% CI)** |
| **PM_2.5_** |  |  |
| Pregnancy | 0.98 (0.94, 1.02) | 0.89 (0.85, 0.93) |
| Trimester 1 | 0.98 (0.94, 1.03) | 0.93 (0.88, 0.98) |
| Trimester 2 | 1.02 (0.97, 1.07) | 0.98 (0.93, 1.04) |
| Trimester 3 | 0.97 (0.93, 1.02) | 0.94 (0.89, 0.99) |
| **NO_2_** |  |  |
| Pregnancy | 1.11 (1.07, 1.16) | 1.19 (1.14, 1.25) |
| Trimester 1 | 1.06 (0.99, 1.14) | 1.10 (1.03, 1.18) |
| Trimester 2 | 0.96 (0.88, 1.06) | 0.97 (0.89, 1.07) |
| Trimester 3 | 1.09 (1.02, 1.17) | 1.13 (1.05, 1.21) |
| **O_3_** |  |  |
| Pregnancy | 1.06 (1.03, 1.10) | 1.11 (1.07, 1.15) |
| Trimester 1 | 1.07 (1.01, 1.14) | 1.13 (1.05, 1.20) |
| Trimester 2 | 0.98 (0.92, 1.05) | 0.99 (0.92, 1.06) |
| Trimester 3 | 1.12 (1.05, 1.19) | 1.18 (1.10, 1.27) |

Estimated odds ratio (ORs) and 95% confidence intervals (CIs) of cerebral palsy for ambient air pollution at per interquartile range (IQR) increase, adjusted for year of birth, season of conception, maternal individual characteristics (age at delivery, race/ethnicity, education level, primary insurance type of prenatal care), zip-code rural-urban commuting code, and census-tract social vulnerability index (total score).

Trimester-specific exposures were co-adjusted in the same model. Single-pollutant model analyzed each pollutant separately, and multiple-pollutant model was modelled by co-adjusting all three pollutants in the same model.

| **eTable 10. Associations between ambient particulate matter with a diameter 2.5μm or smaller (PM_2.5_, in ug/m3), nitrogen dioxide (NO_2_, in ppb), ozone (O_3_, in ppb) during pregnancy and the risk of cerebral palsy, excluded CP cases with postnatal injuries.** | | |
| --- | --- | --- |
|  | **Single pollutant model**  **OR (95% CI)** | **Multiple pollutants model**  **OR (95% CI)** |
| **PM_2.5_** |  |  |
| Pregnancy | 1.00 (0.97, 1.03) | 0.90 (0.87, 0.94) |
| Trimester 1 | 1.00 (0.96, 1.04) | 0.96 (0.92, 1.01) |
| Trimester 2 | 0.99 (0.95, 1.04) | 0.95 (0.90, 1.00) |
| Trimester 3 | 0.99 (0.95, 1.03) | 0.95 (0.91, 0.99) |
| **NO_2_** |  |  |
| Pregnancy | 1.14 (1.10, 1.17) | 1.21 (1.16, 1.26) |
| Trimester 1 | 1.05 (0.99, 1.11) | 1.08 (1.02, 1.15) |
| Trimester 2 | 1.00 (0.92, 1.08) | 1.01 (0.93, 1.10) |
| Trimester 3 | 1.08 (1.01, 1.14) | 1.11 (1.04, 1.18) |
| **O_3_** |  |  |
| Pregnancy | 1.08 (1.05, 1.11) | 1.12 (1.09, 1.15) |
| Trimester 1 | 1.10 (1.04, 1.15) | 1.17 (1.10, 1.24) |
| Trimester 2 | 0.99 (0.93, 1.05) | 0.98 (0.92, 1.05) |
| Trimester 3 | 1.09 (1.03, 1.15) | 1.15 (1.08, 1.22) |

Note: 226 CP cases with records of drowning, automobile, vehicle types other than automobile, and other types of accidents were excluded in the analyses.

Estimated odds ratio (ORs) and 95% confidence intervals (CIs) of cerebral palsy for ambient air pollution at per interquartile range (IQR) increase, adjusted for year of birth, season of conception, maternal individual characteristics (age at delivery, race/ethnicity, education level, primary insurance type of prenatal care), zip-code rural-urban commuting code, and census-tract social vulnerability index (total score).

Trimester-specific exposures were co-adjusted in the same model. Single-pollutant model analyzed each pollutant separately, and multiple-pollutant model was modelled by co-adjusting all three pollutants in the same model.

| **eTable 11. Associations between ambient particulate matter with a diameter 2.5μm or smaller (PM_2.5,_ in ug/m^3^), nitrogen dioxide (NO_2_, in ppb), ozone (O_3_, in ppb), and a chemical mixture during pregnancy and in the negative control period, with the risk of cerebral palsy (results presented in Figure 3).** | | | |
| --- | --- | --- | --- |
|  | **Prenatal exposure,**  **Main model**  **OR (95% CI)** | **Prenatal exposure in**  **NCE analysis**  **OR (95% CI)** | **Negative control exposure in  NCE analysis**  **OR (95% CI)** |
| **PM_2.5_** |  |  |  |
| Pregnancy | 1.00 (0.97, 1.04) | 1.03 (0.97, 1.09) | 0.97 (0.92, 1.02) |
| Trimester 1 | 1.00 (0.96, 1.04) | 1.02 (0.97, 1.07) | 1.01 (0.97, 1.05) |
| Trimester 2 | 1.00 (0.96, 1.05) | 1.01 (0.96, 1.06) | 0.96 (0.91, 1.01) |
| Trimester 3 | 0.98 (0.95, 1.02) | 0.99 (0.95, 1.03) | 1.00 (0.95, 1.04) |
| **NO_2_** |  |  |  |
| Pregnancy | 1.14 (1.10, 1.18) | 1.13 (1.01, 1.25) | 1.01 (0.91, 1.12) |
| Trimester 1 | 1.05 (0.99, 1.12) | 1.10 (0.98, 1.23) | 0.91 (0.82, 1.02) |
| Trimester 2 | 1.00 (0.92, 1.08) | 0.98 (0.87, 1.10) | 1.00 (0.86, 1.16) |
| Trimester 3 | 1.08 (1.02, 1.15) | 1.11 (1.03, 1.22) | 1.02 (0.91, 1.14) |
| **O_3_** |  |  |  |
| Pregnancy | 1.08 (1.05, 1.11) | 1.04 (1.00, 1.09) | 1.05 (1.00, 1.09) |
| Trimester 1 | 1.10 (1.04, 1.15) | 1.10 (0.99, 1.23) | 1.02 (0.94, 1.10) |
| Trimester 2 | 0.99 (0.93, 1.05) | 1.03 (0.94, 1.14) | 0.98 (0.88, 1.09) |
| Trimester 3 | 1.10 (1.04, 1.16) | 1.08 (0.99, 1.17) | 0.97 (0.89, 1.06) |
| **Mixture** |  |  |  |
| Pregnancy | 1.23 (1.17, 1.29) | 1.16 (1.02, 1.31) | 1.06 (0.95, 1.22) |
| Trimester 1 | 1.21 (1.10, 1.34) | 1.24 (1.07, 1.43) | 0.97 (0.85, 1.12) |
| Trimester 2 | 0.95 (0.85, 1.07) | 0.96 (0.85, 1.08) | 0.96 (0.79, 1.16) |
| Trimester 3 | 1.22 (1.10, 1.35) | 1.23 (1.11, 1.36) | 0.98 (0.83, 1.16) |

Estimated odds ratio (ORs) and 95% confidence intervals (CIs) of cerebral palsy for ambient air pollution at per interquartile range (IQR) increase, adjusted for year of birth, season of conception, maternal individual characteristics (age at delivery, race/ethnicity, education level, primary insurance type of prenatal care), zip-code rural-urban commuting code, and census-tract social vulnerability index (total score).

All pollutants were modelled as single exposure. Trimester-specific exposures were co-adjusted in the same model. The NCE analysis co-adjusted for the prenatal exposure and the negative control exposure variables in the same model.

| **eTable 12**. **Mediation analysis of adverse birth outcomes in the associations between ambient air pollution and cerebral palsy.** | | | | | |
| --- | --- | --- | --- | --- | --- |
|  | | Total effect | Natural (pure) indirect effect | Natural (total) direct effect | Percentage mediated |
|  |  | OR (95% CI) | OR (95% CI) | OR (95% CI) | %* |
| PM_2.5_ | |  |  |  |  |
|  | Preterm birth | 1.01 (0.97, 1.03) | 1.02 (1.01, 1.02) | 0.99 (0.95, 1.01) | N/A |
|  | Very preterm birth | 1.01 (1.00, 1.03) | 1.02 (1.01, 1.02) | 0.99 (0.99, 1.02) | N/A |
|  | Low birthweight | 1.00 (0.97, 1.02) | 1.01 (1.01, 1.01) | 0.99 (0.97, 1.01) | N/A |
|  | Small for gestational age | 1.00 (0.98, 1.04) | 1.01 (1.00, 1.01) | 0.99 (0.96, 1.03) | N/A |
| NO_2_ | |  |  |  |  |
|  | Preterm birth | 1.14 (1.10, 1.18) | 1.01 (1.00, 1.01) | 1.13 (1.11, 1.16) | 2.2% |
|  | Very preterm birth | 1.14 (1.11, 1.15) | 1.01 (1.00, 1.01) | 1.14 (1.11, 1.15) | 3.2% |
|  | Low birthweight | 1.13 (1.11, 1.17) | 1.01 (1.01, 1.01) | 1.12 (1.10, 1.16) | 7.5% |
|  | Small for gestational age | 1.14 (1.11, 1.16) | 1.01 (1.01, 1.01) | 1.13 (1.10, 1.15) | 6.2% |
| O_3_ | |  |  |  |  |
|  | Preterm birth | 1.08 (1.02, 1.11) | 1.01 (1.00, 1.01) | 1.07 (1.01, 1.10) | 12.9% |
|  | Very preterm birth | 1.06 (1.04, 1.10) | 1.00 (1.00, 1.01) | 1.06 (1.03, 1.10) | 6.3% |
|  | Low birthweight | 1.07 (1.05, 1.10) | 1.00 (1.00, 1.01) | 1.07 (1.05, 1.09) | 5.9% |
|  | Small for gestational age | 1.08 (1.05, 1.10) | 1.00 (1.00, 1.00) | 1.08 (1.05, 1.10) | 1.5% |

Estimated odds ratio (ORs) and 95% confidence intervals (CIs) of cerebral palsy for ambient air pollution at per interquartile range (IQR) increase, adjusted for year of birth, season of conception, maternal individual characteristics (age at delivery, race/ethnicity, education level, primary insurance type of prenatal care), zip-code level urban rural commuting code, and census-tract social vulnerability index (total score).

*Mediated percentages were only presented when the estimated direct effects and indirect effects had a same direction of association.

| **eTable 13. Associations between ambient air pollution and the risk of cerebral palsy, stratified by child’s sex.** | | | | | |
| --- | --- | --- | --- | --- | --- |
|  | | PM_2.5_ | NO_2_ | O_3_ |  |
|  |  | OR (95% CI) | OR (95% CI) | OR (95% CI) |  |
| Child’s sex | |  |  |  |  |
|  | Females | 1.00 (0.96, 1.05) | 1.13 (1.07, 1.19) | 1.10 (1.06, 1.15) |  |
|  | Males | 1.00 (0.96, 1.05) | 1.15 (1.10, 1.20) | 1.06 (1.02, 1.10) |  |

Estimated odds ratio (ORs) and 95% confidence intervals (CIs) of cerebral palsy for ambient air pollution at per interquartile range (IQR) increase, adjusted for year of birth, season of conception, maternal individual characteristics (age at delivery, race/ethnicity, education level, primary insurance type of prenatal care), zip-code rural-urban commuting code, and census-tract social vulnerability index (total score).

**eTable 14. Associations between ambient air pollution and the risk of cerebral palsy, stratified analyses by maternal neighborhood and individual socioeconomic status.**

| **Socioeconomic characteristics** | | **PM_2.5_** | **NO_2_** | **O_3_** |
| --- | --- | --- | --- | --- |
|  |  | **OR (95% CI)** | **OR (95% CI)** | **OR (95% CI)** |
| **Neighborhood social vulnerability index** | | | | |
| Total score | |  |  |  |
|  | High vulnerability | 0.93 (0.89, 0.99) | 1.14 (1.09, 1.20) | 1.04 (1.00, 1.09) |
|  | Low vulnerability | 1.05 (1.01, 1.09) | 1.15 (1.10, 1.20) | 1.11 (1.07, 1.15) |
| Socioeconomic domain | |  |  |  |
|  | High vulnerability | 0.92 (0.87, 0.97) | 1.16 (1.10, 1.22) | 1.06 (1.02, 1.11) |
|  | Low vulnerability | 1.06 (1.01, 1.10) | 1.14 (1.09, 1.19) | 1.08 (1.04, 1.12) |
| **Individual socioeconomic status** | | | | |
| Maternal race/ethnicity | |  |  |  |
|  | Hispanic of any race | 1.02 (0.97, 1.06) | 1.17 (1.12, 1.22) | 1.09 (1.05, 1.13) |
|  | Non-Hispanic White | 0.99 (0.93, 1.04) | 1.13 (1.06, 1.20) | 1.10 (1.04, 1.16) |
|  | African American/Black | 0.92 (0.81, 1.04) | 0.99 (0.87, 1.13) | 1.00 (0.91, 1.10) |
|  | Asian | 1.11 (0.99, 1.24) | 1.26 (1.13, 1.41) | 0.98 (0.87, 1.09) |
| Maternal education level | |  |  |  |
|  | <12th grade | 0.99 (0.93, 1.05) | 1.16 (1.09, 1.23) | 1.06 (1.01, 1.12) |
|  | High school or diploma | 0.99 (0.95, 1.04) | 1.14 (1.08, 1.19) | 1.08 (1.04, 1.12) |
|  | College and above | 1.06 (0.98, 1.14) | 1.14 (1.06, 1.23) | 1.09 (1.02, 1.17) |
| Maternal insurance type | |  |  |  |
|  | Government | 0.99 (0.95, 1.04) | 1.14 (1.08, 1.19) | 1.08 (1.04, 1.12) |
|  | Private | 1.05 (1.00, 1.10) | 1.18 (1.12, 1.24) | 1.08 (1.03, 1.13) |

Estimated odds ratio (ORs) and 95% confidence intervals (CIs) of cerebral palsy for ambient air pollution at per interquartile range (IQR) increase, adjusted for year of birth, season of conception, and maternal individual characteristics (age at delivery, race/ethnicity, education level, primary insurance type of prenatal care). Social vulnerability index is on census-tract level and ranges from 0 to 1, high vulnerability is defined as score ≥ 75^th^ percentile. If a covariate was stratified upon it is omitted in the covariate adjustment set. All p-values for interactions were larger than 0.05.


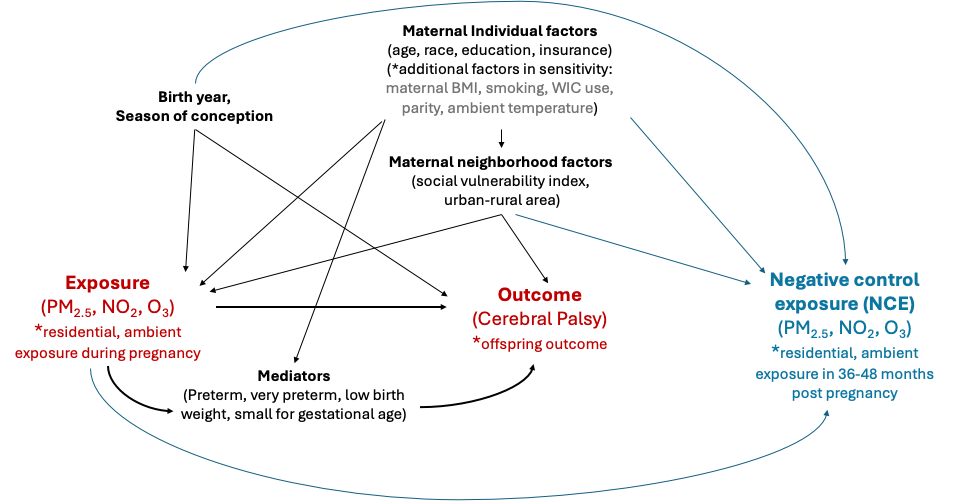


**eFigure 1**. A directed acyclic graph (DAG) of the confounding structure.


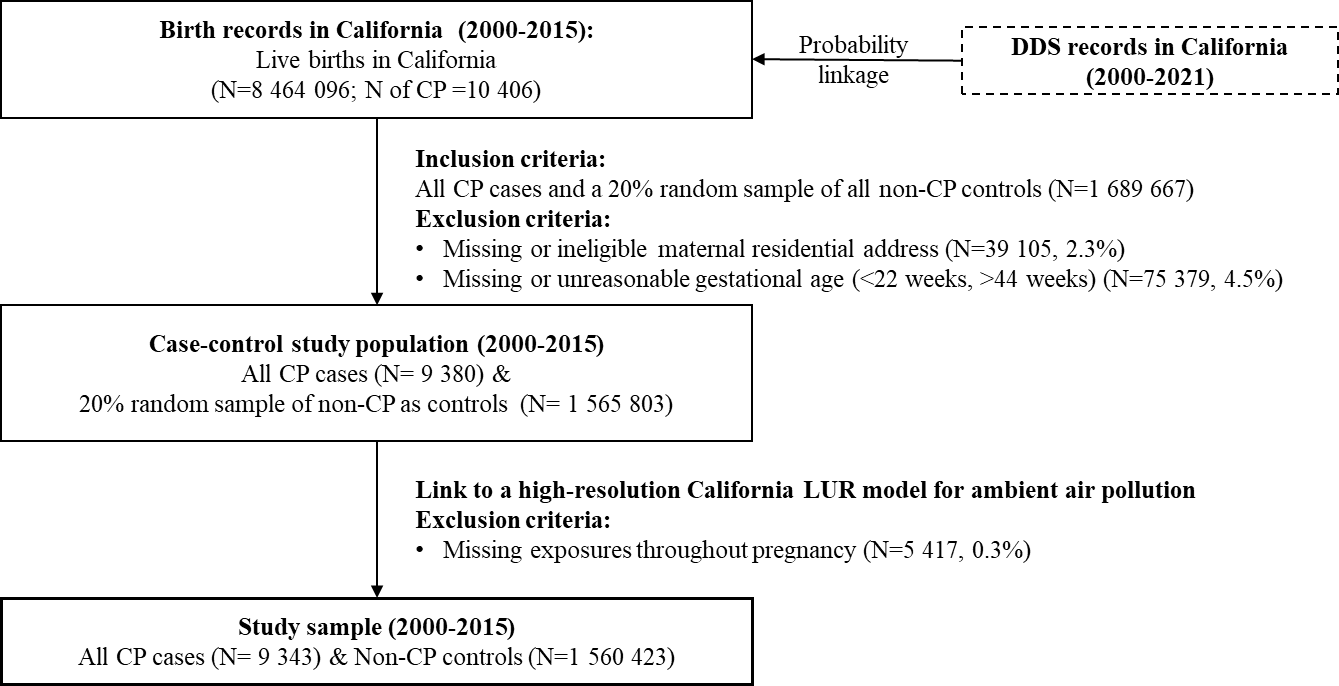


**eFigure 2. Flowchart of the study population**

**
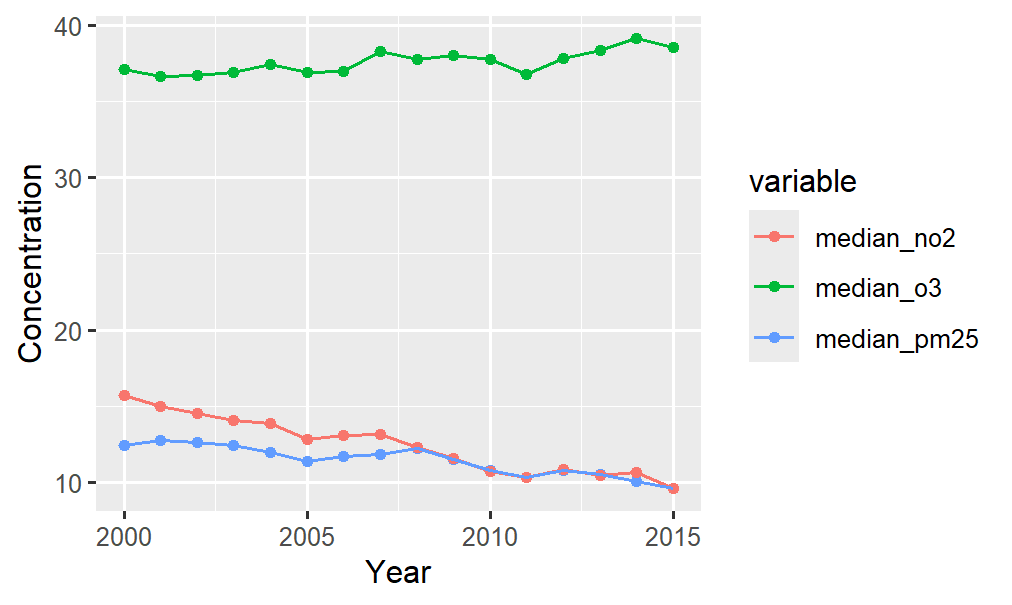
**

**eFigure 3. Median levels of prenatal exposures to particulate matter with a diameter 2.5μm or smaller (PM_2.5,_ in ug/m^3^), nitrogen dioxide (NO_2_, in ppb), and ozone (O_3_, in ppb) across years**
